# 

**Published:** 1887-05

**Authors:** 


### CONTENTS, MAY, '87, (VOL. 2, NO. 11.)

ORIGINAL COMMUNICATIONS—Interstitial Uterine Fibro-Myoma Complicating Delivery. By E. J. Beall, M. D,. Fort Worth, Texas.............................. 447

Lithotomy, Supra-Pubic Operation. By R. Menger, M.D., San Antonio, Texas. ............................... 454

Extraordinary Case of Blood Poisoning. By F. A. Schmitt, M. D., Schulenburg, Texas,........................ 555

CULLINGS FROM CONTEMPORARIES—The Administration of Gaseous Enemata for Pulmonary Phthisis,........... 458

Laparotomy and Intestinal Suture, Dr. Jno. A. Wyeth,.. 462

SOCIETY NOTES—Texas State Medical Association, Nineteenth Annual Meeting; Synopsis of Proceedings,.... 472

EDITORIAL—The President Elect ,Texas State Medical Association, Dr. Sam. R. Burroughs.................. 499

Grateful Acknowledgement.......................... 501

The Recent Meeting of the Tex. State Med. Association. 502

The Reason Why...................................... 603

